# Draft genome of *Escherichia coli* 546 with toxigenic potential, an epizootic important pathogenic isolate obtained from a sick piglet

**DOI:** 10.1128/mra.00081-26

**Published:** 2026-05-18

**Authors:** Alexander S. Tishchenko, Sergey V. Kopyltsov, Artem P. Sedashev, Nadejda L. Machneva, Sviatoslav V. Fedorovich, Anastasia V. Elisiutikova

**Affiliations:** 1 Biotechnology Center, Kuban State Agrarian University named after I.T. Trubilin 64863 , Krasnodar, Russia; 2 Department of Biotechnology, Biochemistry and Biophysics, Kuban State Agrarian University named after I.T. Trubilin 64863 , Krasnodar, Russia; 3 Center for Molecular Genetic Research, Kuban State Agrarian University named after I.T. Trubilin 64863 , Krasnodar, Russia; University of Manitoba, Winnipeg, Manitoba, Canada

**Keywords:** *Escherichia coli*, escherichiosis, exotoxins

## Abstract

The toxigenic *Escherichia coli* strain 546 was isolated from the tubular bone of a 10-day-old sick piglet. Genetic analysis revealed the presence of toxin-encoding genes, indicating the strain’s potential as a component for improving porcine vaccines.

## ANNOUNCEMENT

Due to the role of toxigenic *Escherichia coli* in piglet escherichiosis (1), the identification of toxin genes in an isolated strain suggests its potential as a source of vaccine antigens (2).

The *E. coli* strain 546 was isolated on 30 March 2022 from the bone marrow of the tubular bone of a 10-day-old piglet obtained from the Maxim Gorky CJSC (Kavkazsky District, Krasnodar Region, 45.549404, 40.722303). The strain has the antigenic formula O41:K99 by slide agglutination. It produces thermolabile (LT) and thermostable (ST) toxins, along with hemolysins A, B, C, D, and E. Clinical materials were collected prior to antibiotic and chemotherapy and inoculated onto nutrient media as quickly as possible. Endo agar was used for the primary isolation of bacteria from the family Enterobacteriaceae. Sorbitol-MacConkey agar was employed to distinguish enterohemorrhagic *E. coli* from enterotoxigenic strains. Casitose yeast extract broth was used to cultivate enterobacteria to accumulate biomass, study their growth properties, and isolate LT and ST enterotoxins. All media were from Merck (Germany). Incubation was aerobic at 37°C for 18–24 h. The strain was identified using the ENTEROtest 16 and ENTEROtest 24 systems (Erba Lachema s.r.o., Brno, Czech Republic). The isolate was cultured on Luria-Bertani (LB) agar (Lennox, Laboratorios Conda, Spain). Exotoxins LT and ST were detected by the mouse paw edema and suckling mouse tests, respectively (3–5).

After 24 h of incubation in LB medium at 37°C, the bacterial culture was centrifuged (0.5 mL aliquot; 13,000 rpm, 5 min). DNA was isolated using the DNeasy Blood & Tissue Kit (Qiagen, Germany). The library for whole-genome sequencing was prepared using the Illumina DNA Prep Tagmentation Kit (Illumina, Inc., USA), and the quality and quantity were assessed using a Qubit fluorometer (Thermo Fisher, USA). Sequencing was performed on the MiSeq device, utilizing 2 × 150 bp MiSeq v2 Reagent Kit (Illumina, Inc., USA). Read quality control was performed using FastQC v.0.11.9 (6). Adapters were removed with Trimmomatic v.0.39 (7). *De novo* assembly of the genome was performed using SPAdes v.3.15.3 (8). The quality of the assembly was assessed with CheckM2 v.1.1.0 (9). The genome was annotated using the National Center for Biotechnology Information (NCBI) Prokaryotic Genome Annotation Pipeline (PGAP) (10). ABRicate v1.0.0 (11) was used to screen contigs for resistance (ResFinder) (12) and virulence (VFDB) genes (13). Default parameters were used for all tools unless otherwise specified.

Taxonomic affiliation was determined via digital DNA–DNA hybridization (dDDH) (14) using the Type (Strain) Genome Server (TYGS) (15).

Genome assembly of *E. coli* 546 based on 849,101 reads generated 326 contigs totaling 5,147,652 bp (scaffold N50 = 111,514 bp; GC content = 50.59%; coverage = 54.1×). A total of 5,087 protein-coding sequences were predicted. PGAP annotation of the genome identified 7 rRNAs, 71 tRNAs, 12 noncoding RNAs, 239 pseudogenes, and 6 toxin-encoding CDSs: RTX hemolysins HlyA and HlyE, heat-labile enterotoxin LT (subunits A and B), and heat-stable enterotoxins, ST-I and ST-II. In total, 205 virulence factors were detected. TYGS analysis showed 71.7% average nucleotide identity (ANI) between our strain and *E. coli* DSM 30083 (GCF_000690815.1).

## Data Availability

This Whole Genome Shotgun project has been deposited in GenBank under the following accession numbers: BioSample SAMN31181481, BioProject PRJNA887444, SRA SRR22478783, and GenBank accession number GCA_025817575.1. The version described in this paper is the first version.
